# Preparation and Properties of Highly Transparent SiO_2_ Aerogels for Thermal Insulation

**DOI:** 10.3390/gels8110744

**Published:** 2022-11-16

**Authors:** Baolu Shi, Long Xie, Bin Ma, Zhiliang Zhou, Baosheng Xu, Lijie Qu

**Affiliations:** 1 Institute of Advanced Structure Technology, Beijing Institute of Technology, Beijing 100081, China; 2 Beijing Key Laboratory of Lightweight Multi-functional Composite Materials and Structures, Beijing Institute of Technology, Beijing 100081, China; 3 Beijing Electro-Mechanical Engineering Institute, Beijing 100074, China; 4 Beijing Institute of Spacecraft System Engineering, Beijing 100094, China

**Keywords:** SiO_2_ aerogels, high transparency, thermal insulation, mesoporous materials

## Abstract

SiO_2_ aerogels have attracted extensive attention due to their unique structural characteristics, which exhibit many special properties, especially good optical transparency. As far as we know, the sol-gel stage during the synthesis of aerogel plays an important role in the construction of the gel skeleton. In this study, we adjusted the amount of silicon source and catalyst to explore the best scheme for preparing highly transparent SiO_2_ aerogels, and further clarify the effects of both on the properties of SiO_2_ aerogels. Results indicated that the pore size distribution was between 10 and 20 nm, the thermal conductivity was between 0.0135 and 0.021 W/(m·K), and the transmittance reached 97.78% at 800 nm of the aerogels, better than most studies. Therefore, it has the potential to be used in aerogel glass for thermal insulation.

## 1. Introduction

Aerogel [1] is a typical thermal insulation material with a three-dimensional (3D) porous network structure connected by nanoparticles. It has been widely used in numerous fields due to its many unique characteristics and properties, such as low density, high porosity, extremely low thermal conductivity and excellent acoustic properties. SiO_2_ aerogel [2] with excellent optical transparency has emerged as the most representative aerogel due to its inexpensive cost and straightforward preparation process [3,4,5,6]. Transparent SiO_2_ aerogel [7,8] has been utilized extensively in Cherenkov detectors [9,10], thermal insulation [11,12,13,14,15], energy-saving [13,16,17,18,19], photoelectric materials [20,21,22], wearables fields [23], thermal collectors [24,25,26,27,28], adsorbents and sensors. Thus, transparent SiO_2_ aerogel has great research value and the prospects are very broad.

The thermal insulation performance of aerogels has been demonstrated to be due to the low thermal conductivity provided by the high porosity structure [29] and the pore size is preferably smaller than the air free path (70 nm). In addition, high transparency performance requires that the pores of aerogel be much smaller than the wavelength of visible light, so as to minimize the scattering of block. As a result, the porous size of aerogels with thermal insulation as well as highly transparent must be small and uniformly distributed to avoid large deviation of light at the interface of pores. Moreover, SiO_2_ aerogels are generally prepared by the sol-gel method. Due to the internal disordered porous structure, they are easily cracked and broken during the preparation process, and it is a big challenge to obtain the overall block structure without defects [30]. In most research, the supercritical drying method without gas-liquid interface is used to avoid the generation of capillary forces and reduce the shrinkage of gel.

Researchers have been trying to improve the optical transparency of SiO_2_ aerogels, but it is exceedingly difficult to combine thermal insulation with high transparency [31,32,33,34]. At present, there are many mainstream or mature strategies to improve the transmittance of SiO_2_ aerogels. Firstly, in the sol-gel stage, Shimizu et al. [35] found, based on surfactants, that different concentrations of the base catalyst affected the size and uniformity of aerogels skeleton, and thus affected transparency. Zhao et al. [24] proposed a rapid-hydrolysis-condensation procedure and restricted-cluster, and the molar ratio of catalyst was identified as the most critical parameter in the experimental process in order to obtain highly transparent aerogels. Moreover, during the drying process after sol-gel, by controlling shrinkage in the aging process of the gels, Lei et al. [36] prepared polysesiciloxane aerogels with smaller particles and pores. The aerogels with the density of 0.25 g/cm^3^ had a transmittance of 70% at 550 nm. Similarly, Tabata et al. [9] prepared SiO_2_ aerogels with higher transparency than conventional methods using a method called pinhole drying [37]. Despite the fact that the transmittance was improved and there were no cracks in the appearance of aerogels, the shrinkage rate of aerogels after drying was very high, which was a common problem in atmospheric drying [1,21]. Finally, after the preparation stage, high-temperature annealing is one of the effective methods to change the microstructure of aerogels. Strobach et al. [25,26] used the viscosity change of aerogels at high temperature to continue to improve transparency. The results showed that aerogels were relatively stable at a low temperature, but particles tend to be extremely aggregated and densified at high temperature. Hence, the size of the scattering source also had a great dependence on temperature, resulting in the increase of transparency. Although temperature treatment can significantly improve transparency, high-temperature treatment is time-consuming and laborious, and will cause shrinkage of aerogels, resulting in an increase in thermal conductivity. In general, the monolithic SiO_2_ aerogel system became a promising material for windows with highly energy-efficient [12,14,38].

In this paper, we aim to prepare highly transparent SiO_2_ aerogels with a concise and efficient method. Thus, the alkaline one-step sol-gel method was adopted, and tetramethoxysilane (TMOS), a silicon source with a small molecular volume was selected, and by adjusting the amount of silicon source and alkali catalyst to simply regulated the gel structure. Lastly, the highly transparent SiO_2_ aerogels were obtained by the appropriate drying method. Here, no additional surfactants and post-treatment were required, the SiO_2_ aerogels with great advantages in optical transparency were directly obtained through simple processes. Compared to glass, SiO_2_ aerogels do not generate glare spots, and hold great promise as a means of thermal insulation in the interspace of double-glazing systems as well as in solar collectors.

## 2. Results and Discussion

### 2.1. FTIR Spectrum Analysis

**Figure 1:**
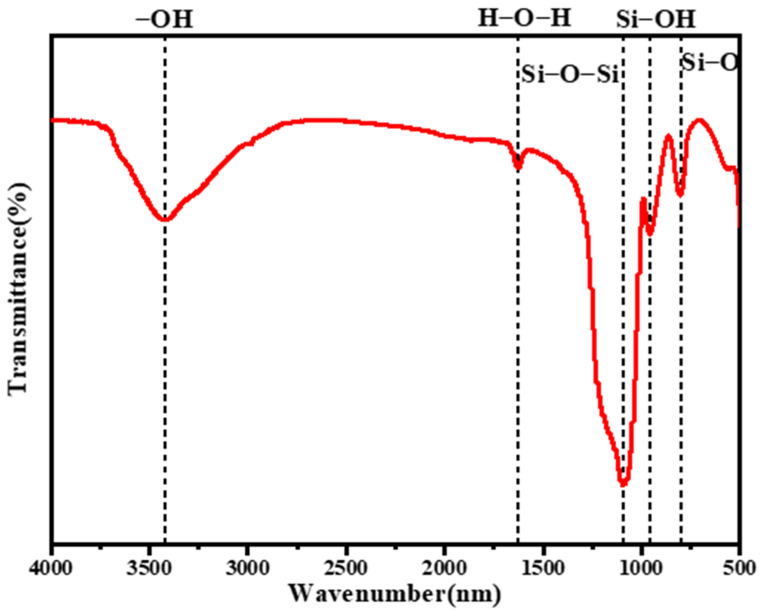
FTIR spectrum of SiO_2_ aerogels.

Figure 1 showed the FTIR spectrum of SiO_2_ aerogels, in which 3422 cm^−1^ was the antisymmetric stretching vibration peak of -OH and 1627 cm^−1^ was the bending vibration peak of H-O-H; 1094 cm^−1^ was the antisymmetric stretching vibration peak of the Si-O-Si group, which belonged to the characteristic peak of SiO_2_, indicating that the main component of sample was SiO_2_; 957 cm^−1^ was the stretching vibration peak of Si-OH, and this group was the main reason for the hydrophilicity of SiO_2_ aerogel; 802 cm^−1^ was the symmetric stretching vibration peak of Si-O, which also belonged to the characteristic peak of SiO_2_.

### 2.2. Microstructure Analysis

**Figure 2:**
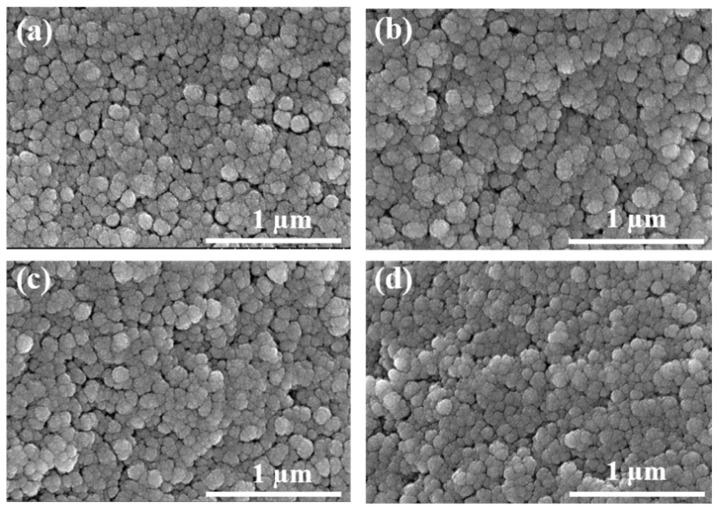
SEM of SiO_2_ aerogels: the concentration of NH_4_OH solution was 0.75 mol/L and the mass fractions of TMOS were (**a**) 15 wt.%; (**b**) 25 wt.%; (**c**) 35 wt.%; (**d**) 45 wt.%.

The micromorphology of SiO_2_ aerogels was a typical nano-porous 3D network structure. As shown in Figure 2, when the concentration of NH_4_OH solution was 0.75 mol/L and the mass fraction of TMOS was 15 wt.%; 25 wt.%; 35 wt.% and 45 wt.%, the micromorphologies with continuous porousity of the prepared SiO_2_ aerogels were very similar. Small and uniform particles were closely connected with each other through the "neck", and the structure was relatively dense. Due to the different mass fractions of TMOS, the aerogels occurred different shrinkage after drying, as shown in Table 1, resulting in a secondary change in the microstructure. With the decrease in the mass fraction of silicon source, the shrinkage gradually increased, and the structures tended to be dense, which made the structure of SiO_2_ aerogels with high and low mass fraction very similar, and still maintained a pearl-like 3D network structure. The increase in TMOS content only deepened the densification of the structure slightly. Therefore, the SiO_2_ aerogels prepared under the condition of different TMOS mass fractions presented a small difference in the micromorphology.

**Figure 3:**
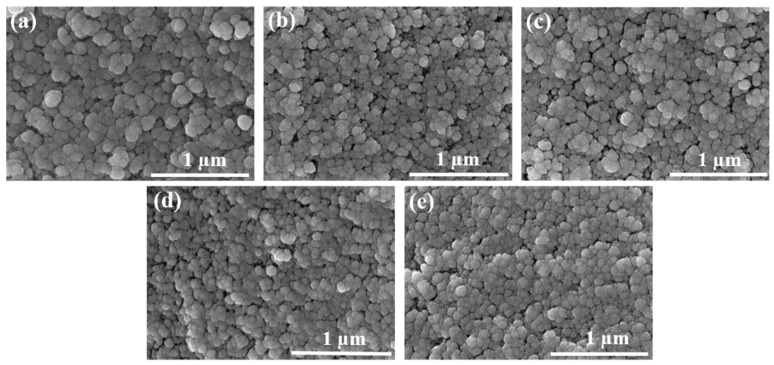
SEM of SiO_2_ aerogels: the mass fraction of TMOS was 45 wt.% and the concentration of NH_4_OH solutions were (**a**) 0.15 mol/L; (**b**) 0.30 mol/L; (**c**) 0.45 mol/L; (**d**) 0.60 mol/L; (**e**) 0.75 mol/L.

However, When the mass fraction of TMOS was 45 wt.% and the concentration of NH_4_OH solution was 0.15 mol/L; 0.30 mol/L; 0.45 mol/L; 0.60 mol/L and 0.75 mol/L, the size of SiO_2_ particles and pores decreased with the increase in the concentration of NH_4_OH solution, and the structure tended to be denser, as shown in Figure 3. It was attributed to the high catalyst concentration improving the speed of gelation, so that the silicon source can rapidly hydrolyze and condense into primary particles. Then, due to the depletion of the silicon source, the formation of secondary particles lacked raw materials at the later stage, so the clustering process stopped abruptly, resulting in smaller SiO_2_ particles, smaller pores, and more uniform distribution [24].

**Figure 4:**
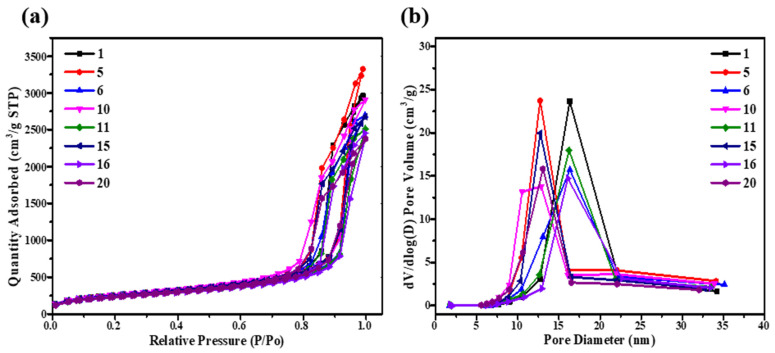
(**a**) N_2_ adsorption-desorption isotherm curves of SiO_2_ aerogels; (**b**) pore size distribution curves of SiO_2_ aerogels.

Moreover, as shown in Figure 4, the effect of silicon source content and catalyst concentration on the microstructure was also illustrated. Figure 4a showed the N_2_ adsorption-desorption isotherm curves of SiO_2_ aerogels which belonged to the type IV, a typical characteristic of mesoporous materials [21]. With the increase in TMOS mass fraction, the specific surface area gradually decreased. On the contrary, with the increase in NH_4_OH solution concentration, the specific surface area increased accordingly. This was mainly because more silicon sources, the hydrolysis and condensation reaction will be more sufficient, and more SiO_2_ particles can be generated to secondary particles, as well as the catalyst enabling the reduction of SiO_2_ particle size by rapid gel process. As shown in Figure 4b, it can be seen that the pore size distribution of each sample was mainly concentrated between 10 and 20 nm, which was divided obviously into two concentrated points due to the different concentration of NH_4_OH solution. When the concentration of NH_4_OH solution was low, it was between 15 and 20 nm with wide distribution, and when the concentration of NH_4_OH solution was high, it is between 10 and 15 nm with narrow distribution. However, the pore size of the NH_4_OH solution with the same concentration and different TMOS content was roughly the same. Therefore, the effect of NH_4_OH solution concentration on the pore size distribution was more significant.

### 2.3. Thermal Properties

**Figure 5:**
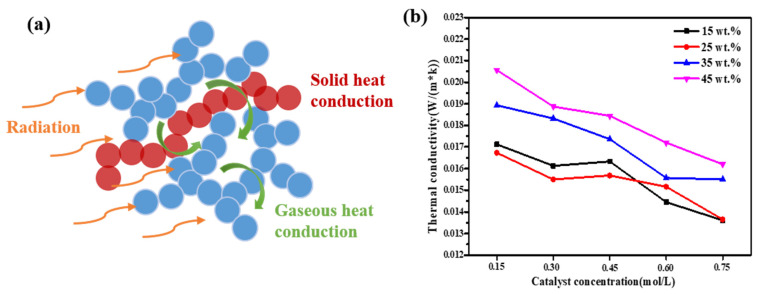
(**a**) The heat transfer modes in aerogels; (**b**) thermal conductivity curves of SiO_2_ aerogels.

As shown in Figure 5a, there are three ways of heat conduction in aerogels: solid-phase heat transfer, gas convection heat transfer, and radiation heat transfer. In general, radiation heat transfer is ignored at room temperature [1]. Figure 5b showed the thermal conductivity of SiO_2_ aerogels. It can be seen that with the increase in TMOS mass fraction, the thermal conductivity increased, because higher TMOS mass fraction led to higher bulk density, resulting in an increase in the solid-phase heat conduction of the network skeleton. However, the thermal conductivity of 15 wt.% aerogels was slightly higher than that of 25 wt.%. Maybe because too little TMOS content led to larger pores, resulting in higher gas convection heat transfer. At the same time, when the mass fraction of TMOS was same, the thermal conductivity decreased with the increase in the concentration of NH_4_OH solution. This was owing to a high catalyst concentration, which led to smaller SiO_2_ particles and pores, which limited the solid-phase heat transfer and gas convection heat transfer, resulting in a lower final thermal conductivity.

**Figure 6:**
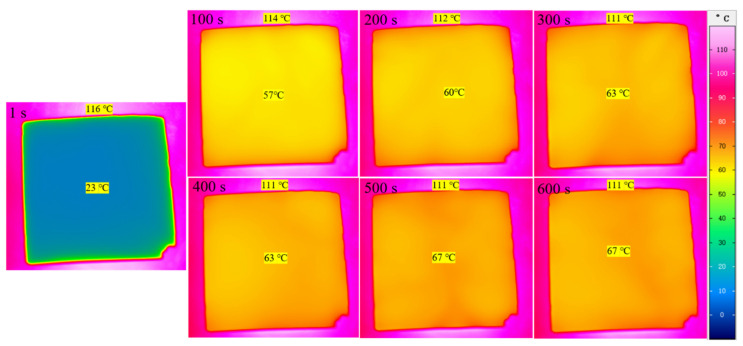
The infrared thermal imaging of the SiO_2_ aerogel.

Figure 6 showed the infrared thermal imaging of the SiO_2_ aerogel. It can be seen that the surface temperature of the SiO_2_ aerogel tended to be stable after 300 s, and the temperature difference ΔT = 44 °C at 600 s, which was about 40% of panel temperature, indicating that the highly transparent SiO_2_ aerogel had excellent thermal insulation performance.

Table 2 listed the thermal conductivities of SiO_2_ aerogels, xerogel and cryogel. Through comparison, we found that thanks to the supercritical drying method, SiO_2_ aerogel had the lowest thermal conductivity and had a great advantage in thermal insulation.

### 2.4. Optical Transmittance

According to the Rayleigh-Gans theory, the extinction coefficient of aerogels is proportional to the bulk density of aerogels and the diameter of the scattering source [7], as follows:

(1) $$\sigma_{s}=4\pi^{4}\frac{\rho_{ap}}{\rho_{SiO_{2}}}\frac{d^{3}}{\lambda^{4}}\left(\frac{n^{2}-1}{n^{2}-2}\right)$$

where $\sigma_{s}$ is scattering coefficient, $\rho_{ap}$ is apparent density or bulk density of SiO_2_ aerogels, $\rho_{SiO_{2}}$ is true density of amorphous silicon (2.2 g/cm^3^), d is diameter of particle scattering center, λ is wavelength, n is relative refractive index (n_1_ is refractive index of the SiO_2_ particles, n_2_ is refractive index of the external medium (air)). The formulation applies to isolated SiO_2_ spheres, which approximate a highly porous bulk.

In detail, one of the scattering sources is the macroscopic defects of appearance, which may be caused during the sol-gel process or demolding process [16], so the whole preparation process should try to ensure no pollution and destruction. Another scattering source is the scattering of nano-porous structures, which is the result of the interaction between SiO_2_ particles and pores [16]. In fact, we can control the density of SiO_2_ aerogels and the diameter of the scattering source, but because the relationship between the diameter of the scattering source and the extinction coefficient is a power of 3, it becomes a more critical factor.

**Figure 7:**
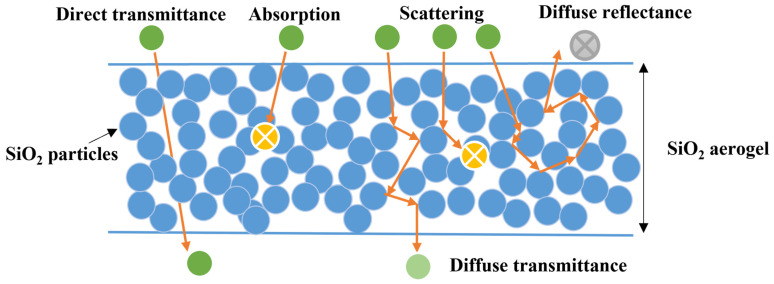
The propagation paths of light in SiO_2_ aerogels.

As shown in Figure 7, SiO_2_ aerogels themselves have a very low absorption of light. When SiO_2_ aerogels are irradiated by a beam of light, it may interact with the scattering source in the aerogels and then weaken the intensity of transmittance. According to the Rayleigh-Gans theory, the smaller the size of the SiO_2_ aerogels structural unit, the smaller the extinction coefficient and the higher the transmittance [41]. Therefore, the optical transparency of SiO_2_ aerogels was related to the gel structure construction [41,42]. The optimization of composition and microstructure control are effective strategies to improve the high performance and multi-performance of SiO_2_ aerogels [1].

**Figure 8:**
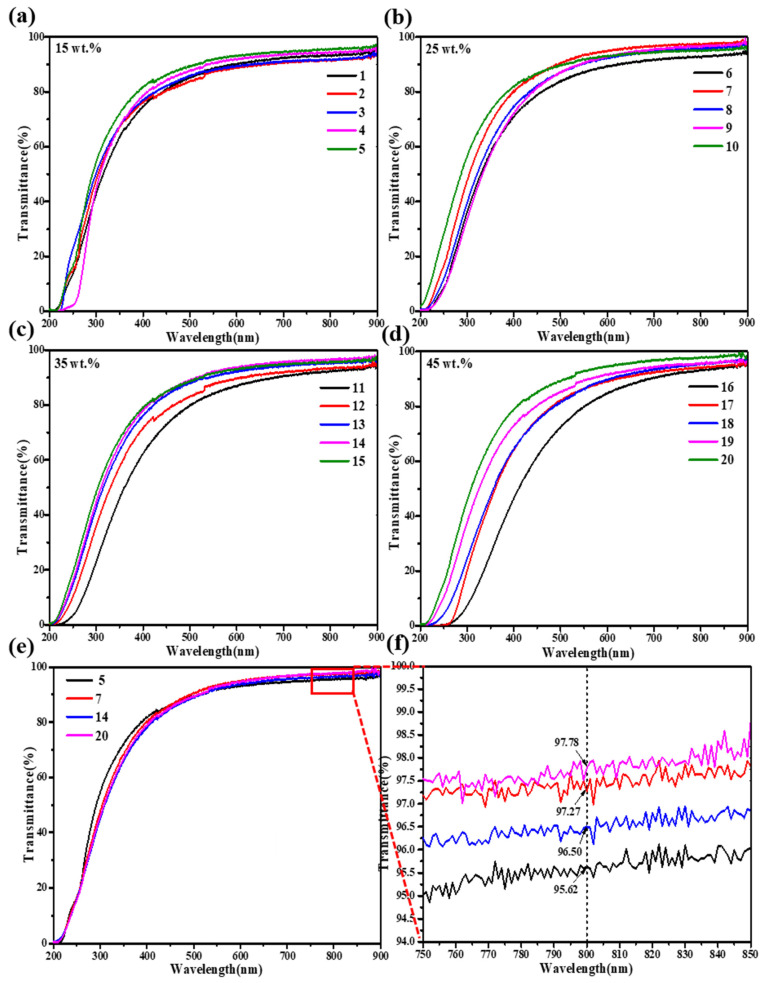
Transmittance curves of SiO_2_ aerogels with different TMOS mass fractions: (**a**) 15 wt.%; (**b**) 25 wt.%; (**c**) 35 wt.%; (**d**) 45 wt.%; (**e**) the highest transmittance among the TMOS mass fractions of 15–45 wt.% and its (**f**) local magnification of wavelengths from 750 nm to 850 nm.

Figure 8 showed that the transmittance curves of SiO_2_ aerogels. Figure 8a–d were the transmittance curves under the same TMOS mass fraction and different NH_4_OH solution concentrations, respectively. According to Figure 8e,f, the results showed that the transmittance of SiO_2_ aerogels prepared by 45 wt.% TMOS and 0.75 mol/L NH_4_OH solution were the highest, which reached 97.78% at 800 nm. The reason for that was because the high content of silicon source led to sufficient SiO_2_ particles generated, while the high concentration of catalyst promoted the hydrolysis and condensation reaction of SiO_2_, and the cluster was restricted. Under these conditions, the generated SiO_2_ particles and pores were small and uniform, the scattering source was small, which reduced the deviation of light as well as the scattering of light in the aerogels, so the transparency of aerogels was the highest. The results were consistent with the previous analysis.

## 3. Conclusions

We used different mass fractions of TMOS and different concentrations of NH_4_OH solution to simply control the structure of SiO_2_ aerogels, thereby improving the transmittance. The micromorphology including densification, particle size and pore size distribution of SiO_2_ aerogels were closely related to the silicon source content and catalyst concentration. After adjusting the experimental parameters, the particles and pore size distribution of SiO_2_ aerogels were small, and the thermal conductivity was low. More notably, the highest transmittance of SiO_2_ aerogels can reach 97.78% at 800 nm, which was higher than that of ordinary SiO_2_ aerogels and undoubtedly broaden the application prospect of SiO_2_ aerogels in highly transparent materials, such as aerogel glass. However, the mechanical properties and hydrophilicity of highly transparent SiO_2_ aerogels should be further modified for improvement.

## 4. Materials and Methods

### 4.1. Materials

TMOS (AR) was purchased from Shanghai Aladdin Biochemical Technology Co., Ltd. (Shanghai, China). Methanol (AR) was purchased from Beijing Tongguang Fine Chemical Company (Beijing, China). The water (deionized water) was purchased from Shanghai Yien Chemical Technology Co., Ltd. (Shanghai, China). NH_4_OH (GR) was purchased from Shanghai Maclin Biochemical Technology Co., Ltd. (Shanghai, China). Ethanol (AR) was purchased from Beijing Tongguang Fine Chemical Company (Beijing, China). All chemical regents were used without further purification.

### 4.2. Preparation of SiO_2_ Aerogels

**Figure 9:**
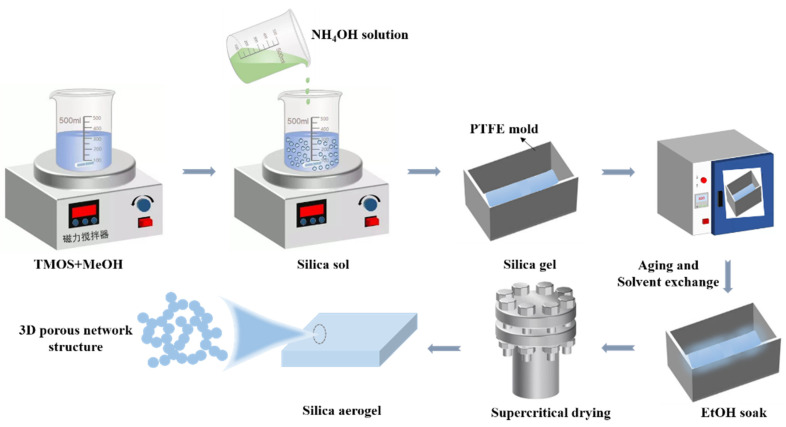
Schematic of preparation process of highly transparent SiO_2_ aerogels.

**Figure 10:**
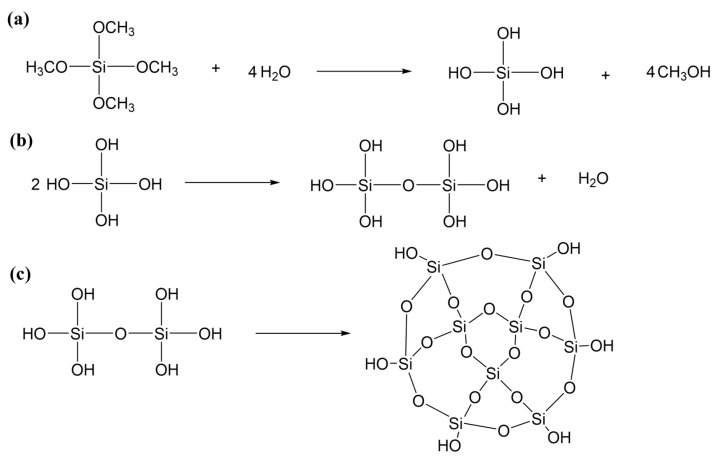
(**a**) Hydrolysis reaction of TMOS; (**b**) condensation reaction of TMOS after hydrolysis; (**c**) further cross-linking by condensation reaction to form a 3D network.

**Figure 11:**
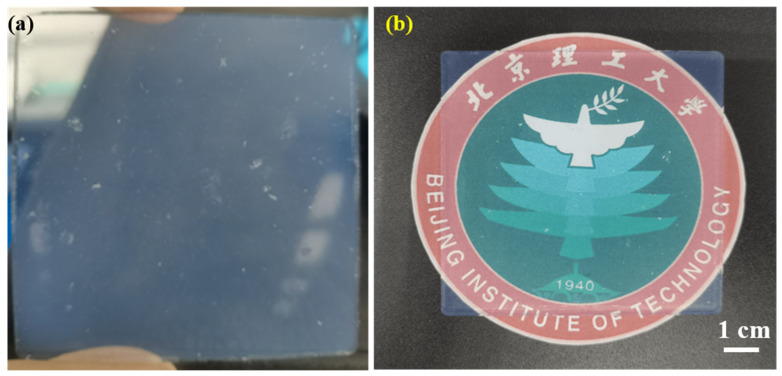
Pictures of SiO_2_ aerogels with difference transparency: (**a**) sample 12 and (**b**) sample 20.

The schematic of the preparation process was shown in Figure 9 and the reaction process was shown in Figure 10. The preparation formulations of SiO_2_ aerogels were listed in Table 3. In the experiments, the mass ratios of TMOS were 15, 25, 35 and 45 wt.%, and the concentrations of NH_4_OH solution were 0.15, 0.30, 0.45, 0.60 and 0.75 mol/L. Firstly, a certain amount of TMOS and methanol were mixed at room temperature and stirred for 30 min. Then, NH_4_OH solutions were added and continued to stir until uniform and moved to the mold, which needed to be sealed and placed in a 30 °C oven for 24 h. After the demolding process, the gel was soaked in ethanol for 3 days for solvent replacement and cleaning. Finally, supercritical CO_2_ drying [43] was performed to obtain highly transparent SiO_2_ aerogels. The specific parameters of supercritical CO_2_ drying were set as follows: the temperature of the drying kettle was 32 °C, the temperature of the separating kettle was 37 °C, the pressure of the storage tank was 5.5 MPa, the drying pressure was 7.5 MPa, the separation pressure was 5.5 MPa, and the supercritical state was maintained for about 1 h. Parameters can be adjusted according to different aerogel formulations. Figure 11 showed the pictures of the prepared SiO_2_ aerogels with difference transparency.

### 4.3. Characterizations

The microstructure of SiO_2_ aerogels was observed using a Hitachi S4800 scanning electron microscope. Nitrogen adsorption-desorption tests for observation of pore structure were performed using a Micrometeritics APSP 2460 automatic specific surface area and porosity analyzer. The DRPL-III high-precision thermal conductivity tester was used to test the thermal conductivity of the SiO_2_ aerogels; the temperature of the hot surface was 40 °C, the temperature of the cold surface was 20 °C, and the pressure was 5 N. A TU-1901 double-beam UV-Vis spectrophotometer was used to perform spectral scanning on the SiO_2_ aerogels to obtain the transmittance (T%), and the test wavelength range was 200–900 nm. The SiO_2_ aerogels were formed into a size of 80×80 mm and the thickness was 2–3 mm.

## Figures and Tables

**Table 1:** Shrinkage of SiO_2_ aerogels with different mass fractions of TMOS.

TMOS (wt.%)	15	25	35	45
Line Shrinkage (%)	22~27	11~15	8~10	5~9

**Table 2:** The thermal conductivities of SiO_2_ aerogels, xerogel and cryogel [29,30,39,40].

Samples	SiO_2_ Aerogel	SiO_2_ Xerogel	SiO_2_ Cryogel
Thermal conductivity (W/(m·K))	0.01–0.03	0.03–0.09	0.03–0.05

**Table 3:** Preparation formulations of highly transparent SiO_2_ aerogels.

Samples	TMOS (wt.%)	TMOS (g)	Methanol (g)	NH_4_OH (g)	Deionized Water (g)
1	15	2.47	10.4	0.019	3.6
2				0.038	
3				0.058	
4				0.077	
5				0.097	
6	25	4.67	10.4	0.019	3.6
7				0.038	
8				0.058	
9				0.077	
10				0.097	
11	35	7.53	10.4	0.019	3.6
12				0.038	
13				0.058	
14				0.077	
15				0.097	
16	45	11.45	10.4	0.019	3.6
17				0.038	
18				0.058	
19				0.077	
20				0.097	

## Data Availability

Data are available from the authors. Samples of the compounds are available from the authors.
